# Endocrine regulation of predator-induced phenotypic plasticity

**DOI:** 10.1007/s00442-014-3102-8

**Published:** 2014-10-05

**Authors:** Stuart R. Dennis, Gerald A. LeBlanc, Andrew P. Beckerman

**Affiliations:** 1 Department of Animal and Plant Sciences, University of Sheffield, Western Bank, Sheffield, UK; 2 Environmental and Molecular Toxicology Program, Department of Biological Sciences, North Carolina State University, Raleigh, NC 27695-7633 USA

**Keywords:** Phenotypic plasticity, *Daphnia pulex*, Juvenile hormone, Gene expression, Developmental control, Predation risk

## Abstract

Elucidating the developmental and genetic control of phenotypic plasticity remains a central agenda in evolutionary ecology. Here, we investigate the physiological regulation of phenotypic plasticity induced by another organism, specifically predator-induced phenotypic plasticity in the model ecological and evolutionary organism *Daphnia pulex*. Our research centres on using molecular tools to test among alternative mechanisms of developmental control tied to hormone titres, receptors and their timing in the life cycle. First, we synthesize detail about predator-induced defenses and the physiological regulation of arthropod somatic growth and morphology, leading to a clear prediction that morphological defences are regulated by juvenile hormone and life-history plasticity by ecdysone and juvenile hormone. We then show how a small network of genes can differentiate phenotype expression between the two primary developmental control pathways in arthropods: juvenoid and ecdysteroid hormone signalling. Then, by applying an experimental gradient of predation risk, we show dose-dependent gene expression linking predator-induced plasticity to the juvenoid hormone pathway. Our data support three conclusions: (1) the juvenoid signalling pathway regulates predator-induced phenotypic plasticity; (2) the hormone titre (ligand), rather than receptor, regulates predator-induced developmental plasticity; (3) evolution has favoured the harnessing of a major, highly conserved endocrine pathway in arthropod development to regulate the response to cues about changing environments (risk) from another organism (predator).

## Introduction

Phenotypic plasticity—the expression of different phenotypes in different environments by single genotypes—can alter the mean and variance of traits on which selection can act. This topic draws the attention of ecologists interested in the origins and consequences of trait variation, of evolutionary biologists interested in plasticity as a source of selectable variation, and of developmental biologists interested in genes, hormones and the developmental control of traits such as morphology and life history (Nijhout 2003b; Pigliucci 2001; Sultan 2007; Tollrian and Harvell 1999). Identifying the role of development and physiology in the expression of plasticity is central to defining the mechanisms that underpin trait variation. Strong links between phenotypic plasticity and developmental biology are established in plants, where it is understood how endocrine physiology mediates environmental signals to produce phenotypes. Plant biology long ago embraced a molecular physiology–ecology agenda to link population and physiological and molecular ecology (e.g. Baldwin et al. 2001; Weston et al. 2008). In contrast, developmental and physiological explanations for phenotypic plasticity in animals are much less frequent and less developed (for a recent review, see Flatt and Heyland 2011).

There are several animal examples detailing how endocrine signalling can mediate single trait responses to abiotic features of the environment (Table 1). Classic examples centre on arthropods and their two major developmental hormones—juvenile hormones and ecdysteroids. They include examples of endocrine control of polymorphism such as seasonal colour variation in butterfly wings (Rountree and Nijhout 1995) and temperature regulation of colour morph in *Manduca sexta* (Truman et al. 1973). They also include examples of the endocrine control of threshold traits (on–off; polyphenism) as examples of plasticity including butterfly wing patterning (Brakefield et al. 1998), sexual ornaments in horned beetles (Emlen and Nijhout 1999) and temperature-dependent sex determination in *Daphnia* (Olmstead and LeBlanc 2007). The central feature of these examples is a response of the juvenoid and ecdysteroid hormone signalling pathways to an abiotic environmental stress, producing change in a single trait. These examples (Table 1) provide valuable evidence that polymorphisms and polyphenisms are under endocrine control. Here, we extend such efforts to reveal endocrine control of phenotypic plasticity in response to another organism—the case of predator-induced phenotypic plasticity.

**Table 1:** Examples of endocrine-mediated polyphenisms (trait), the identified mechanisms and their environmental cues

Organism	Polymorphism	Mechanism	Cue	References
Butterflies	Hindwing melanism and eyespot size	Ecdysteroid timing and duration	Seasonal: photoperiod Temperature	Beldade and Brakefield (2002), Brakefield et al. (1998), Endo and Kamata (1985), Rountree and Nijhout (1995), Sawada et al. (2002)
	Eyespot presence	Ecdysteroid receptor expression	Seasonal: photoperiod Temperature	
*Manduca sexta* larvae	Larval colour green/black	JH titre	Temperature	Safranek and Riddiford (1975), Suzuki and Nijhout (2006)
*Onthophagus* beetles	Horn growth	Ecdysteroid pulse	Body size	Emlen and Nijhout (1999), Moczek and Emlen (1999)
	Horn size	JH titre		
*Daphnia* spp.	Sex determination	JH titre	Seasonal: photoperiod Temperature Nutrition Pop density	Hebert (1978), Olmstead and Leblanc (2002), Tatarazako et al. (2003)
Crickets	Wing length	JH titre	Temperature Photoperiod Diet Pop density	Zera and Bottsford (2001), Zera and Denno (1997)
		Ecdysteroid titre		
Termites	Caste differentiation	JH titre	Pheromone	Hartfelder and Emlen (2005), Zhou et al. (2007)
Aphids	Winged forms (alates)	JH titre	Seasonal: photoperiod Temperature Pop density	Hardie (1980), Hardie et al. (1985)

Predator-induced plasticity (i.e. predator-induced defences) has emerged across many taxa as an example of continuous phenotypic plasticity, despite years of being considered an on–off, threshold trait (Roff 2002). The plasticity is typically detected in three classes of traits: morphology, life history and behaviour. The ecological responses of freshwater vertebrates and invertebrates to predator cues have been well studied (e.g. Beckerman et al. 2010; Dennis et al. 2011; Hammill et al. 2008; Hoverman and Relyea 2009, 2001b; Riessen 1999; Tollrian 1995b; Tollrian and Harvell 1999). Key findings from cladocera and rotifera (for reviews see Lass and Spaak 2003; Tollrian and Dodson 1999), and anuran and odonate species (e.g. Laurila et al. 2002; Relyea 2001a; Van Buskirk 2002) indicate that predator-induced defences are continuous, multivariate, adaptive, confer a survival benefit, alter the distribution of populations and species, and can substantially influence population dynamics. The reaction norms for these induced defences (responses) are also rarely as steep as a simple polyphenism definition would suggest (see Dennis et al. 2011; Hammill et al. 2008).

The predator-induced variation in the timing of the life cycle and morphology that defines predator-induced plasticity indicates that it is likely a function of variation in the temporal and spatial regulation of development. This system thus offers one of the richest possibilities for extending our understanding about how endocrine physiology mediates developmental control of phenotypic plasticity, extending previous efforts limited to single trait responses to abiotic stress (Table 1). Here we provide a functional explanation of how predator-induced phenotypic plasticity is generated.

Specifically, we show that predator-induced prey morphological defences in *Daphnia pulex* are regulated in a dose-dependent manner by the juvenoid hormone signalling pathway, which, along with ecdysteroids, form the major endocrine signalling pathways in most arthropods. Specifically, we show that evolution has favoured the harnessing of a major, highly conserved endocrine pathway in arthropod development to regulate the response to cues about changing environments (risk) from another organism (predator).

We present three sets of data central to this result. First, we present a concise and novel synthesis of predation and the physiological regulation of arthropod somatic growth and morphology, leading to a clear prediction that morphological defences are regulated by juvenile hormone and life-history plasticity by ecdysone and juvenile hormone. Second, we present a small gene network dominated by highly conserved nuclear receptors (Escriva et al. 2004) that can, via RT-qPCR, be used to distinguish between activity of the juvenile hormone and ecdysone signalling pathways under experimental conditions. Finally, we present experimental data on gene expression using this network to clearly show that predator-induced prey morphological defences in *Daphnia pulex* are regulated in a dose-dependent manner by the juvenile hormone pathway.

## Methods

Our methods consist of four steps. First we document, via a synthetic review of the arthropod developmental biology literature, that inducible defences in arthropods must be under hormonal control by the juvenoid or ecdysteroid hormone signalling pathway. In contrast to recent work focusing on neurotransmitters (Weiss et al. 2012), we focus our study on endocrine regulation. Environmental signals received by sensory apparatus are transmitted, usually via neurotransmitters and the nervous system, to the endocrine system, which then orchestrates appropriate phenotypic responses in target tissues. The effective integration of all these processes is common and important but the endocrine system is always required for phenotypic plasticity in life history and morphology, whereas neurotransmitters alone are sometimes sufficient for behavioural change.

Second, via the same arthropod development literature, we document a small gene network capable of distinguishing, via RT-qPCR, the activity of either pathway under experimental conditions. Third, we use this network via RT-qPCR and experimental exposure of daphnids along a gradient of predation pressure to reveal which pathway is mediating the plastic response to predation risk.

### Study system

**Fig. 1:**
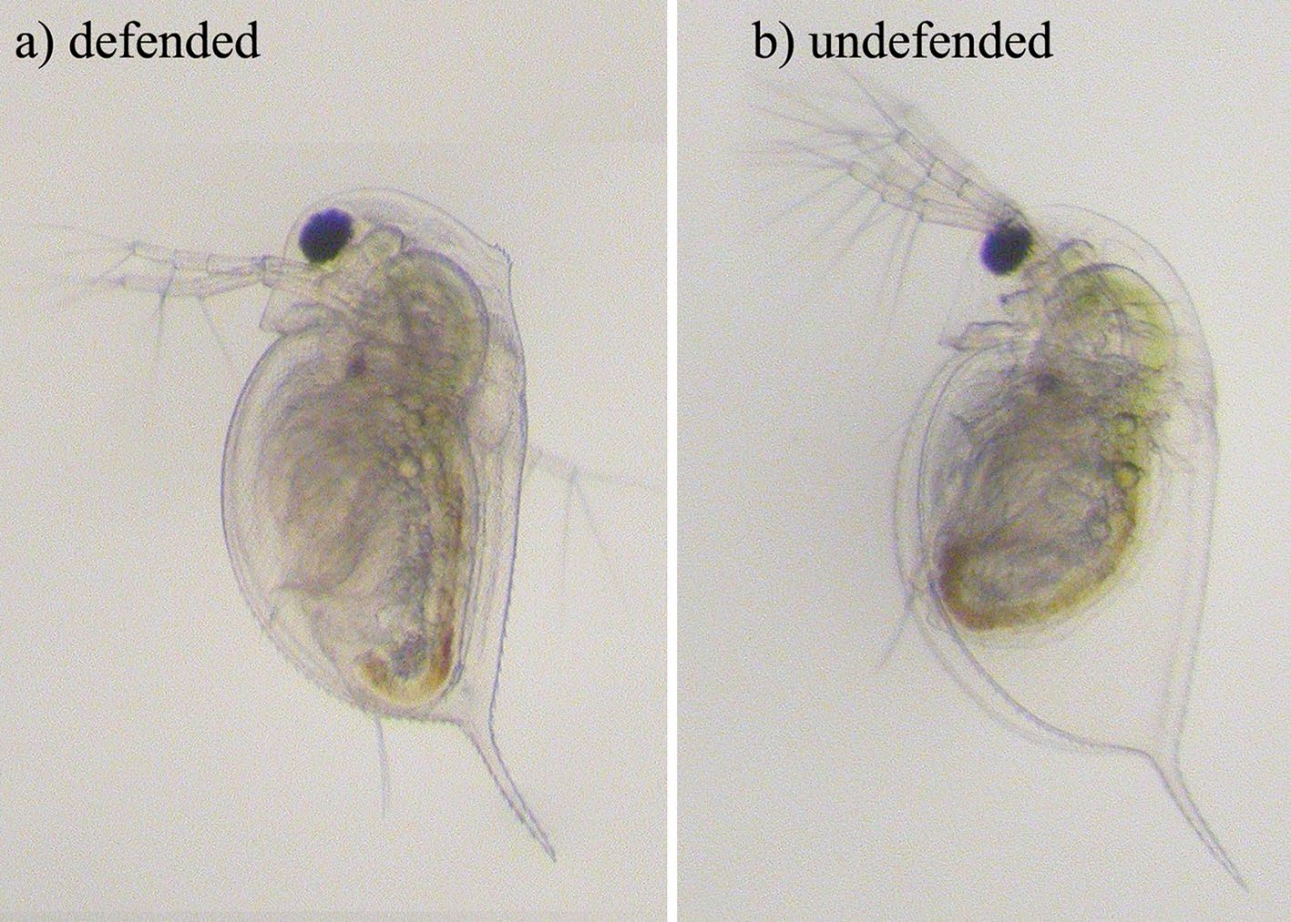
Exposure to chemical cues from midge (*Chaoborus flavicans*) larvae results in a defended phenotype in *Daphnia pulex*. **a** Second-instar daphnid exhibiting a defended morphology. **b** An undefended second-instar daphnid

We use *Daphnia pulex*, a model organism for ecological interactions, ecotoxicology, arthropod developmental biology and environmental genomics (Colbourne et al. 2011). It is a common freshwater crustacean (water flea) that experiences predation risk from the phantom midge *Chaoborous flavicans*. The midge predator produces a low molecular weight kairomone that induces changes in *D. pulex* life history and morphology (Tollrian and Von Elert 1994). Specifically, *C. flavicans* induces later age and larger size at maturity as well as prominent neckteeth during the second and third instars of development, corresponding to the age (size) classes most sensitive to midge predation (see Fig. 1 and Tollrian 1995a). There is extensive genetic variation in these responses (i.e. genetic variation in plasticity), and the responses are adaptive (Beckerman et al. 2010; Hammill et al. 2008; Tollrian 1995b). For example, the induced neckteeth can increase survival by up to 45 % (Hammill et al. 2008). These ecological patterns and decades of research make *D. pulex* a model ecological example.

### Physiological review and discovery of the gene network

We investigated the arthropod literature on the regulation of somatic growth and morphology by juvenoid and ecdysteroid signalling in arthropods. These data provide detailed information on how the regulation of development by the endocrine system may determine patterns of growth, development and morphology under predation risk. These data also identify a gene network capable of distinguishing, via RT-qPCR, the activity of either pathway under experimental conditions.

### Kairomone exposure to reveal juvenoid or ecdysteroid control

In order to discriminate between juvenoid and ecdysteroid control of predator-induced plasticity using the gene network, *D. pulex* were exposed to predator chemical cues along a gradient of increasing predation risk and serially sampled to isolate RNA.

Daphnids were routinely maintained in hard artificial pond water (ASTM 2007), and fed the algae *Chlorella vulgaris* at 21 °C in controlled-temperature rooms on a 16:8 light cycle. We extracted kairomone from frozen *Chaoborus flavicans* (Honka, Germany), following the method developed by Tollrian (see also Hammill et al. 2008; Tollrian 1995b).

To produce a gradient of predation risk, we exposed replicate individuals independently to four concentrations of extracted predator cue (0, 0.1, 0.5, 1 µL mL^−1^).

**Fig. 2:**
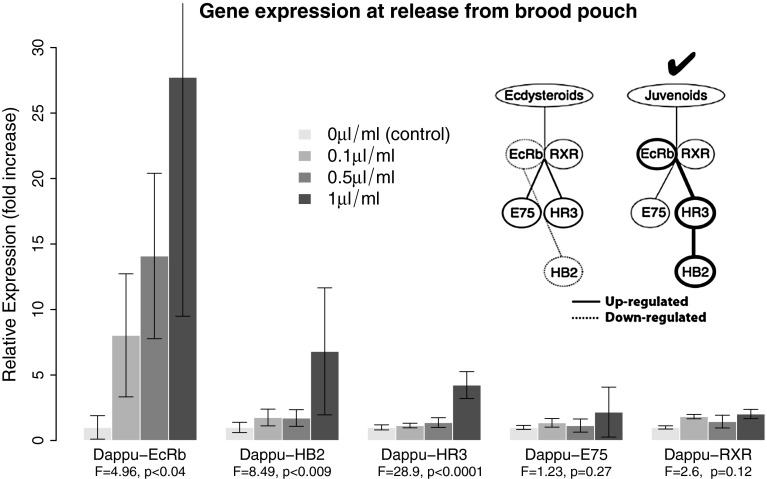
Relative expression of the five genes in the proposed mini gene network. Significant predator kairomone dose-dependent upregulation of Dappu-EcRb, Dappu-HR3 and Dappu-HB2 expression are consistent with an increase in juvenoid hormone titres. *Inset:* a five-gene network, dominated by nuclear receptors central to arthropod development, that can discriminate between activity in the ecdysteroid and juvenoid endocrine pathways. *Solid lines* indicate upregulation (more expression) and *dashed lines* indicate downregulation (less expression). *Thicker* vs. *thinner lines* indicate relative (qualitative) magnitudes of gene expression

For each treatment, 60 third-generation mothers who had finished their second brood were each exposed to the relevant concentration of chemical cue. Exposures began when third-brood embryos developed eyespots (approximately 24 h prior to release from the brood chamber). Each jar was checked hourly, and neonates (10–15) were collected at the time of brood pouch release. Neonates from three sets of 20 mothers for each treatment were pooled in RNALater for subsequent RNA extraction. We focused on expression at brood release because it is in the middle of the established perinatal time course of induced morphological change: embryo exposure to predator kairomones results in substantial morphological expression of neckteeth (Fig. 2a) on a pedestal at instar two, a time delay of ~3–4 days, with release of neonates from the brood pouch being in the middle (Laforsch and Tollrian 2004; Naraki et al. 2013; Parejko 1992).

### RNA isolation and cDNA conversion

Samples were homogenized and RNA isolated from the homogenate using the SV Total RNA Isolation System (Promega), following the manufacturer’s instructions. RNA yield and purity were determined by absorbance (260 nm and 260/280 nm ratio, respectively) using a Nanodrop ND-1000 spectrophotometer. RNA was reverse-transcribed to cDNA with random hexamers and oligo DT primers using the ImProm-II Reverse Transcription system (Promega) and/or the HighCap RT kit (Applied Biosystems), following the manufacturer’s instructions.

### Primer design

Genes were identified via our review and synthesis (see below). Primers for β-actin, EcRb, RXR, E75, HR3 and HB2 were designed using Primer3 and Amplify3X (v3.1.4) after locating EST libraries of the genes from wFleabase. Primers were developed as follows. Blast searches against the genes from other organisms and contigs were assembled using CAP3. Contigs were then blasted against the *Daphnia pulex* genome. The resulting exon–exon spanning primers were: β-actin forward (f)-TGGTCAGGTCATCACCATTG, reverse (r)-CTCGTGGATACCGCAAGATT; DappuEcRb f-TCGTCATCTCGGTCATGTGT, r-TGAACTACCCTCCGAAGACG; DappuRXR f-GTTCAAGAGGAGAGGCAACG, r-AATCACTGGTGGCATCCATATC; DappuE75 f-CACTGGTTCCAATTGCTTTG, r-GTCTCGATCGTAACGTCTTGC; DappuHR3 f-GGGCGTCCATTATGGAGTCA, r-CGGAAGAAACCCTTGCAGC; DappuHB2 f-CAAAGTCCTCCTCCCAAGC, r-CTGTTGGGCAACGTCAACTA.

rt-QPCR (quantitative real-time PCR) was performed (in procedural triplicate) with an Applied Biosystems Step-One real-time PCR machine using default parameters. Amplification mixtures consisted of 2.5 µL SYBR Green PCR Master Mix (Applied Biosystems), 10 nM primers, and 250 ng template cDNA in a total volume of 5 µL. Reactions were held at 95 °C for 10 min, followed by 40 cycles of 95 °C for 15 s followed by 60 °C for 1 min. At the end of cycling, the melting temperatures of PCR products were determined to ensure no amplification of non-target DNA. The comparative CT method (Cy0) was used to assess the relative levels of EcRb, E75, HB2, HR3 and USP normalized to mRNA levels of β-actin measured with the same sample.

## Results

### Endocrine control of predator-induced plasticity: a mini-review and synthesis

There are several independent lines of published evidence that, when combined, predict that predator-induced phenotypic plasticity in morphology and life history is under endocrine regulation by juvenoid and ecdysteroid signalling. The evidence comes from detailed information on why development matters under predation risk and how the endocrine system regulates growth, development and morphology in arthropods. In the following paragraphs we offer a concise review of this evidence.

#### Why development matters under predation risk

Life history (e.g. size and age at maturity) and morphology (e.g. defence structures) are two of the three most common classes of traits that respond to predation risk (the third is behaviour). In vertebrates and invertebrates, adaptive changes of >±10 % in the size and age at maturity and growth rate are not uncommon (Abrams and Rowe 1996; Benard 2004; Peckarsky et al. 2002; Relyea 2001a; Teplitsky et al. 2005). In arthropods (i.e. moulting organisms), such changes in the timing of the life cycle can only occur by shifting the number of, or the duration between, moults.

There are also particularly dramatic responses to predation risk where predator chemical cues induce de novo morphological changes in either shape, colour or the appearance of protuberances (Hoverman and Relyea 2009; Lass and Spaak 2003; Relyea 2001a, b; Tollrian 1995b). The same prey species can often show increases and decreases in size at maturity, age at maturity and growth rate, depending on whether they are exposed to large- or small-sized selective predators (Beckerman et al. 2010).

Such changes in the timing of maturation/metamorphic events and in morphology require major alternations of the physiological processes that regulate somatic growth and development—processes that are well understood in vertebrates and invertebrates. In arthropods (the focus of this study), the timing and spatial patterning of development are regulated by the endocrine system and specifically by the juvenoid/ecdysteroid signalling pathway (see Flatt and Heyland 2011).

#### Endocrine mediation of growth, development and morphology in arthropods

Evidence from a wide range of arthropod species indicates that two hormones oversee the developmental regulation of the timing of moults and the inter-moult duration as well as the transition from juvenile to reproductive adult: juvenile hormone and ecdysteroids (e.g. Cruz et al. 2003; Davidowitz and Nijhout 2004; Flatt and Heyland 2011; Gelman et al. 2002; Riddiford 1993; Riddiford et al. 2003; Truman and Riddiford 2007). Furthermore, research on major morphological changes in insects identifies juvenile hormones as the major regulating factor (e.g. Berger and Dubrovsky 2005; Chang et al. 1993; Hall 1999), either directly as a transcriptional regulator of target genes or indirectly through suppression or augmentation of ecdysteroid-dependent transcriptional regulation (Berger and Dubrovsky 2005; Jindra and Riddiford 1996). Essentially, existing data underpin and spawn our hypothesis that morphological responses to predators in arthropods should be under the control of juvenile hormone, and that life-history responses to predators should be under the control of the interplay between ecdysteroid regulation of the moult and juvenile hormone limitation of maturation. This established developmental detail, combined with knowledge of the continuous nature of predator-induced phenotypic plasticity in *D. pulex* and other arthropods, suggest the following formal prediction: kairomones—external cues of risk from predators—alter phenotypes by mobilising and mediating ecdysteroid and juvenoid hormone and hormone receptor expression in a dose-dependent manner, facilitating fine-scale, adaptive developmental changes in phenotype to predation risk.

### How to discriminate between juvenoid and ecdysteroid signalling

The previous section defined our hypothesis that predator-induced phenotypic plasticity in arthropods should be controlled by the juvenoid and/or ecdysteroid signalling pathways. The literature on arthropod developmental control also reveals that gene expression patterns in a small gene network (Fig. 2), dominated by nuclear receptors, can discriminate between whether the juvenoid or ecdysteroid pathway is correlated with phenotypic plasticity. In addition to supplying a formal tool for investigating physiological regulation of plasticity, we show below that this gene network can also help identify the developmental control mechanism (i.e. changes in receptor or ligand expression profiles, see Nijhout 1999) underpinning this activity.

This network (Fig. 2) comprises five genes (referenced here to the *Daphnia pulex* genome by the prefix “Dappu-”) with orthologues throughout the arthropods: the two components of the nuclear receptor heterodimer for ecdysteroids, Dappu-EcR and Dappu-RXR, and the “early” response genes Dappu-HR3, Dappu-E75 and Dappu-HB2 (a haemoglobin gene). Four of these five gene products (the exception being HB2) are essential for moulting and metamorphosis in* Tribolium* (Tan and Palli 2008) and probably all arthropods (Erezyilmaz 2011; Heyland et al. 2011; King-Jones and Thummel 2005).

Figure 2 shows endocrine pathway specific expression patterns for ecdysteroid or juvenoid action. We derived these pathway-specific patterns of expression from the following detail. Upregulation of HR3 indicates involvement of the ecdysteroid and/or juvenoid pathways without discrimination between them, as both pathways elicit a positive response (Hannas and LeBlanc 2010). In several taxa, E75 is unresponsive to juvenoids but upregulated by ecdysteroid pathway activation (Hannas and LeBlanc 2010; Soin et al. 2008). Moreover, E75 expression is coincident with ecdysteroid pulses in many other insects (King-Jones and Thummel 2005; Sullivan and Thummel 2003), is known to cycle its expression in response to ecdysteroids during the moult cycle in both insects and crustaceans (Priya et al. 2010; Siaussat et al. 2004), and is a repressor of HR3 activity (Hannas et al. 2010). Its precise role during moulting in daphnids remains unclear, but we include it in our profiling due to its regulation of HR3 activity (Hannas et al. 2010).

In *Tribolium*, the haemoglobin gene HB2 is upregulated by juvenoids and downregulated by ecdysteroids (Parthasarathy and Palli 2009). Similarly, HB2 expression can be induced by juvenoids in both *D. magna* and *D. pulex* (Dm-HB2 and Dappu-HB2, respectively) (Rider et al. 2005). If the same pattern (downregulation by ecdysteroids) is true of Dappu-HB2, expression changes in Dappu-HB2 would provide discrimination between the two (juvenoids and ecdysteroids), with the caveat that oxygen tension may also alter the haemoglobin dissociation curve (Lambertsen et al. 1952) and therefore expression of Dappu-HB2. In this context, therefore, Dappu-HB2 may only be an appropriate indicator for laboratory studies. In *D. magna*, EcRb (Dm-EcRb; Dm = *D. magna*) is downregulated by ecdysteroids and upregulated by juvenoids, while expression of its heterodimeric partner (Dm-RXR) is unaffected by either class of hormone. In insects, juvenoid mimics (e.g. methoprene) affect expression of EcRb variably: expression is marginally upregulated at time points distal to ecdysis and downregulated by juvenoids near ecdysis (see Parthasarathy and Palli 2009).

These details combine to allow the gene expression pattern based discrimination of developmental pathways (Fig. 2). They provide a diagnostic tool for experimental evaluation of how environmental stress such as predation is captured by developmental processes to generate phenotypic plasticity. In the next two sections, we use this gene network to reveal the juvenoid pathway regulation of predator-induced plasticity.

### Gene expression and pathway identification

We exposed *D. pulex* to a gradient of four midge kairomone concentrations (control + three concentrations; see “Methods”) and used RT-qPCR to assess expression pattern in the Fig. 2 gene network along this gradient. The discriminatory power of our network revealed which hormone pathway likely regulates predator-induced plasticity: juvenoids.

At brood release, we detect a correlated and very clear, predator-cue, concentration-dependent pattern of relative gene expression consistent with juvenile hormone action (Fig. 2). The same four kairomone concentrations also produce a sigmoid and increasing expression of morphological defence at second instar (see Dennis et al. 2011). Three genes, Dappu-EcRb, Dappu-HR3 and Dappu-HB2, are upregulated in a kairomone-dose-dependent manner relative to control conditions. The remaining two genes, Dappu-RXR and Dappu-E75, are neither up- nor downregulated. This pattern of expression is most coincident with the juvenoid expression profile (Fig. 2, inset). Our conclusion is made more robust by the absence of Dappu-E75 up- and downregulation.

## Discussion

By combining an experiment with *D. pulex* exposed or not exposed to *Chaoborus* kairomone with expression profiles from a small gene network capable of discriminating between developmental control hormone action, we have shown that juvenile hormone signalling is strongly implicated in predator-induced plasticity in a predator cue, dose-dependent manner (Fig. 2).

Our data demonstrate that an environmental signal (kairomone) received during embryonic development is able to co-opt juvenoid signalling for de novo production of a defensive structure. Juvenoid control of morphological change is well established in many arthropods (e.g. Cruz et al. 2003; Davidowitz and Nijhout 2004; Gelman et al. 2002; Riddiford 1993; Riddiford et al. 2003; Truman and Riddiford 2007). Decades of insect research shows that the titre of juvenile hormone influences the phenotype in the next developmental stage.

Several workers have shown that juvenoids can influence daphnid morphology. Oda and colleagues (2011) exposed *D. galeata* to increasing concentrations of methyl farnesoate and fenoxycarb (pesticide juvenile hormone analogue), both of which generated allometric shifts in the relationship between head shape and body size. Furthermore, Miyakawa and colleagues (Miyakawa et al. 2013) have shown that JH analogues may change the perception of the cue, but they and we (Dennis and Beckerman, unpublished data) find no evidence that JH can directly induce neckteeth de novo in *D. pulex*. This indicates that the cue for de novo defence production is not the hormone (JH/MF), but the cue triggers variation in the endocrine control of morphology, presumably via JH interaction.

As we noted in the “Introduction”, changes in the timing of life-cycle events and morphology, hallmarks of predator-induced plasticity, require major alternations of the physiological processes that regulate somatic growth and development—processes that are well understood in vertebrates and invertebrates. Decades of fundamental physiological research have shown that the juvenoid and ecdysteroid hormones and signalling pathways are responsible for such regulation in the arthropods (Flatt and Heyland 2011; Riddiford 2008; Riddiford et al. 2003). Furthermore, evidence from vertebrates and nematodes such as *C. elegans* suggest that there may be similar core developmental pathways regulating development in most animals. This offers the tantalising insight that there are, at least in the arthropods, core and quite conserved developmental processes that interface with the internal and external environment to shape development (Flatt and Heyland 2011) and thus facilitate phenotypic plasticity, which is so often centred on variation in the timing of life-cycle events and morphology. As Flatt and Heyland (2011) reveal throughout their book, linking core developmental processes to peripheral cellular and physiological processes will provide the insight necessary to understand sources of variation and potentially targets of selection in life history and morphology.

### Developmental control of predator-induced plasticity

The patterns of expression in the gene network (Fig. 2) further allow us to reconstruct details about the developmental control mechanism for this dose-dependent effect of juvenile hormone. Developmental biologists suggest that changes in somatic growth and development in insects is controlled by variation in four features of endocrine regulation (Nijhout 1999, 2003a, b): the titre, threshold, timing and sensitive period. The titre is the amount of a given signal (e.g. ligand: hormone/peptide). The effect of this titre depends on whether the amount exceeds a threshold. The action of the hormone involves not only surpassing a threshold, but doing so with timing that overlaps with a sensitive period defined by the receptor. An adjustment or change in any one of these four endocrine traits, as generated by an external cue, represents a mechanism of developmental control and a method by which adaptive plasticity may arise in response to an environmental cue (Nijhout 1999, 2003b). Furthermore, the amount of overshoot of the threshold by the titre, the accuracy of the timing and sensitive period, and the titre of the receptor provide mechanisms by which the phenotypic plasticity can be characterised as a smooth reaction norm, rather than a discrete threshold trait.

Our data show a predator cue generated dose-dependent response in the major components of the juvenoid expression pathway. Combined with previous data on dose-dependent morphological profiles (see Dennis et al. 2011; Hammill et al. 2008), our data indicate that the developmental control mechanism resides in a change in the titre of juvenoids, not a change in the threshold, timing or sensitive period. Specifically, in insects, E75 expression is coincident with ecdysteroid pulses (King-Jones and Thummel 2005); because Dappu-E75 expression is neither up- nor downregulated compared to controls, we can eliminate ecdysteroid titre changes between control and predator environments. Similarly, as Dappu-RXR expression is unchanged, the EcR/RXR receptor expression appears unchanged; the upregulation of Dappu-EcRb is likely a result of juvenoid hormone exposure, especially as neonates are far from ecdysis at this time (see above). The upregulation of Dappu-HR3 and Dappu-HB2 also suggest juvenoid-like titre activity.

### RT-qPCR and the detection of endocrine function

Our gene network reference tool, comprising highly conserved nuclear receptors, offers a compelling alternative to more common methods of assaying hormone titres directly (e.g. radio-immunoassays), and one that has many benefits. First, the amount of biological and consumable material necessary to query the gene network is much less than the amount of haemolymph and consumables needed to use the most advanced tools for radio-immunoassays (RIA), HPLC, mass spectroscopy, etc. Importantly, the low biological material requirements make it possible to query patterns in very small arthropods, which do not typically provide sufficient material for traditional methods. Second, the gene network provides a much more direct route to making inferences about developmental control mechanisms (see above), augmenting typical inferences from RIA and HPLC methods. To gain similar insight into developmental control mechanisms, RIA methods would require assays of multiple genotypes, each with different levels of phenotypic expression, as these methods only measure titres. While exploring variation in multiple genotypes is valuable in its own right, our approach indicates that it is not needed to be able to makes inferences about the mechanisms of developmental control.

### The future of target gene approaches in the genomic era

Rapid advances in multi-omics approaches will soon supersede single/few-target techniques such as RT-qPCR and panel approaches such as microarrays. Falling costs and high-capacity “next-generation sequencing” mean that already it is economically viable and practical to capture an organism’s entire transcriptome simultaneously using RNAseq, rather than a select few targets or microarray. This transcriptome may at first seem attractive, and indeed from a data-generation perspective it is very attractive, but there are many pitfalls, and we advise a degree of caution in the use of such data-dense methods. First, there is a naïve assumption that all parts of the genome will be sequenced with the same probability, governed only by their initial starting abundance, and that that abundance will be proportional in the sequenced dataset. Unfortunately, we already know that this is simply not true (Finotello et al. 2014; Lauss et al. 2013; Tuller 2014). Whereas RT-qPCR ensured “equality” through reaction optimization by designing specific (exon–exon junction spanning) primers for transcripts of interest, and matching amplification efficiencies, amplicon length and GC content, whole-transcript suffers from inherent biases in sequencing due to transcript length (Oshlack and Wakefield 2009), composition and overrepresentation of highly abundant transcripts (Young et al. 2010).

Second, genomes are rich with tens of thousands of genes, all of which can covary in concert and may depend on complicated coexpression and redundancy. Further, small changes in receptor occupancy (as little as 10 %) can have profound biological effects, or none. Therefore, subtle changes in expression that are biologically meaningful or gross changes in expression without biological significance can be incorrectly estimated, especially when many thousands of tests are conducted: a biologically significant relationship may be found or missed purely by chance.

Together, these sequencing biases and type I and II errors combine to suggest a set of tools that are required when using genomic data to ask the types of questions we focus on here. Researchers must be aware of ever-improving statistical techniques that account for (known) sequencing biases (e.g. Beissbarth and Speed 2004; Finotello et al. 2014; Kanehisa et al. 2008; Young et al. 2010; Zeeberg et al. 2003). Researchers must synthesize and use existing biological knowledge, often from multiple disciplines. Then, using a data-dense resource of whole-transcript sequencing in a post hoc directed examination of (a) target genes of known biological function (in a classical qPCR style approach) with (b) functional group analysis (e.g. gene enrichment data), great progress can be made in understanding genomic effects. However, we emphasize that it is essential to have a fundamental understanding of the biological processes of interest. That is not to say that whole-transcript studies are without merit; on the contrary—they are of great importance, especially when used as a library to be probed for explicit questions or to discover novel transcripts. However, we cannot stress enough the fundamental need to understand the biology under investigation.

### Summary

We have presented and evaluated here a developmental control hypothesis for the regulation and expression of predator-induced phenotypic plasticity. Predator-induced morphological plasticity is regulated by ligand dose-dependent juvenoid signalling in *D. pulex*, and, based on our review of arthropod developmental biology, this may be quite a conserved process. We suggest that this, and the logical extension to joint juvenoid/ecdysteroid regulation of life history, is a general mechanism underpinning predator-induced plasticity in arthropods.

First, where changes in life history and morphology are present, and drawing on decades of arthropod endocrine–moulting–morphology research, our eco-devo hypothesis (Sultan 2007) predicts the regulation of predator-induced changes in morphology by the juvenoid endocrine pathway and of life history by a combination of ecdysteroid and juvenoid pathways. Second, we have presented a small gene network dominated by nuclear receptors that are vital for arthropod development and can discriminate experimentally between juvenoid and ecdysteroid action. Third, our endocrine pathway hypothesis is easily linked to established developmental control mechanisms, facilitating further prediction about the physiological regulation of plasticity and potentially the physiological axis on which genetic variation in plasticity is manifest.

Our results confirm a central role of the crustacean juvenile hormone (methyl farnesoate) in the induction of morphological change in juvenile instars of *D. pulex*. We show that the juvenoid pathway, as predicted, appears to regulate induced morphological defences, the central induced phenotypic change early in life in *D. pulex*. We also show that the developmental control of the juvenoid response is centred on the titre of gene products driving the expression. Our results, drawn from the expression of highly conserved genes in one of the most central endocrine signalling systems in arthropods, are likely to be generalisable to other species. The results of this experiment complement previous research into abiotic forms of stress (see Table 1) and reveal juvenile hormone to be central to processing stress.
